# Rescuing ethanol photosynthetic production of cyanobacteria in non-sterilized outdoor cultivations with a bicarbonate-based pH-rising strategy

**DOI:** 10.1186/s13068-017-0765-5

**Published:** 2017-04-14

**Authors:** Zhi Zhu, Guodong Luan, Xiaoming Tan, Haocui Zhang, Xuefeng Lu

**Affiliations:** 1 Key Laboratory of Biofuels, Qingdao Institute of Bioenergy and Bioprocess Technology, Chinese Academy of Sciences, No. 189 Songling Road, Qingdao, 266101 China; 2 Shandong Provincial Key Laboratory of Energy Genetics, Qingdao Institute of Bioenergy and Bioprocess Technology, Chinese Academy of Sciences, No. 189 Songling Road, Qingdao, 266101 China; 3 Shandong Provincial Key Laboratory of Synthetic Biology, Qingdao Institute of Bioenergy and Bioprocess Technology, Chinese Academy of Sciences, No. 189 Songling Road, Qingdao, 266101 China; 4 University of Chinese Academy of Sciences, Beijing, 100049 China; 5 School of Chemical Engineering and Technology, Tianjin University, Tianjin, 300072 China

**Keywords:** Bioethanol, *Synechocystis* sp. PCC6803, Outdoor cultivation, Biocontamination control, pH-rising strategy, Bicarbonate-based Integrated Carbon Capture System

## Abstract

**Background:**

Ethanol photosynthetic production based on cyanobacteria cell factories utilizing CO_2_ and solar energy provides an attractive solution for sustainable production of green fuels. However, the scaling up processes of cyanobacteria cell factories were usually threatened or even devastated by biocontaminations, which restricted biomass or products accumulations of cyanobacteria cells. Thus it is of great significance to develop reliable biocontamination-controlling strategies for promoting ethanol photosynthetic production in large scales.

**Results:**

The scaling up process of a previously developed *Synechocystis* strain Syn-HZ24 for ethanol synthesis was severely inhibited and devastated by a specific contaminant, *Pannonibacter phragmitetus*, which overcame the growths of cyanobacteria cells and completely consumed the ethanol accumulation in the cultivation systems. Physiological analysis revealed that growths and ethanol-consuming activities of the contaminant were sensitive to alkaline conditions, while ethanol-synthesizing cyanobacteria strain Syn-HZ24 could tolerate alkaline pH conditions as high as 11.0, indicating that pH-increasing strategy might be a feasible approach for rescuing ethanol photosynthetic production in outdoor cultivation systems. Thus, we designed and evaluated a Bicarbonate-based Integrated Carbon Capture System (BICCS) derived pH-rising strategy to rescue the ethanol photosynthetic production in non-sterilized conditions. In lab scale artificially simulated systems, pH values of BG11 culture medium were maintained around 11.0 by 180 mM NaHCO_3_ and air steam, under which the infection of *Pannonibacter phragmitetus* was significantly restricted, recovering ethanol production of Syn-HZ24 by about 80%. As for outdoor cultivations, ethanol photosynthetic production of Syn-HZ24 was also successfully rescued by the BICCS-derived pH-rising strategy, obtaining a final ethanol concentration of 0.9 g/L after 10 days cultivation.

**Conclusions:**

In this work, a novel product-consuming biocontamination pattern in cyanobacteria cultivations, causing devastated ethanol photosynthetic production, was identified and characterized. Physiological analysis of the essential ethanol-consuming contaminant directed the design and application of a pH-rising strategy, which effectively and selectively controlled the contamination and rescued ethanol photosynthetic production. Our work demonstrated the importance of reliable contamination control systems and strategies for large scale outdoor cultivations of cyanobacteria, and provided an inspiring paradigm for targeting effective solutions.

**Electronic supplementary material:**

The online version of this article (doi:10.1186/s13068-017-0765-5) contains supplementary material, which is available to authorized users.

## Background

The increasing global environment pollution and potential energy crises have promoted the development of green fuel sustainable production routes to supplement and replace fossil fuels [1, 2]. As the first commercial biofuel product, bioethanol has been widely accepted and used as a gasoline or petroleum alternative or additive [3–5]. At present, a majority of bioethanol is produced through biorefinery processes with sugar-rich agricultural biomass as raw materials; however, this route was controversial due to the dependence on food competitive feedstock [6, 7]. Non-food carbohydrates, represented by lignocellulose, could provide abundant fermentation feedstock for bioethanol production; however, the economic competitiveness was severely restricted by the cost and energy required for pretreatments and enzymatic hydrolysis processes of the raw materials [8]. Comparing with the traditional biorefinery routes based on fermentation processes, ethanol photosynthetic production by recycling CO_2_ and utilizing solar energy has shown great potentials to be a more efficient and sustainable solution [9–11].

Cyanobacteria were a group of photosynthetic autotrophic microorganisms, with simple structures, high photosynthetic efficiency, rapid growths, and convenient genetic manipulations, and thus were seemed as a promising chassis for photosynthetic production [12–14]. Modifications of the natural genetic backgrounds or introductions of artificial metabolic pathways in diverse cyanobacteria have enabled photosynthetic production of types of biofuels and biochemicals [15]. Ethanol was the first reported and most representative biofuel product in cyanobacteria [16, 17]. Ever since the first reported ethanol-synthesizing cyanobacteria cell factory, lots of efforts have been made to enhance ethanol producing capacities in engineered cyanobacteria, improving ethanol titers from 0.46 to 5.5 g/L, with a productivity of 212 mg/L/day [17–20]. Despite comprehensive and systematic research and optimization on strain and cultivation process in laboratory scale, the industrialization of cyanobacteria-based ethanol photosynthetic production was still severely constrained by the lack of mature outdoor cultivation techniques and systems.

Similar to microalgae cultivations, biological contaminations were one of the essential challenges retarding the scaling-up processes of cyanobacteria cultivations, both for natural strains or engineered strains with heterologous pathways [21–24]. As for non-sterilized outdoor cultivations, cellular biomass and target products of the engineered cyanobacteria or microalgae strains were usually inhibited or devastated by types of predators, pathogens, and parasite [21–23]. In our efforts to scale up the ethanol photosynthetic production with a previously developed *Synechocystis* strain Syn-HZ24 [19, 20], biological contaminations were also the main threat. Ethanol accumulations in outdoor non-sterilized cultivation systems were usually completely consumed by at least one specific invading contaminant microorganism. To accomplish efficient and stable ethanol photosynthetic production with cyanobacteria in large scale, strict control systems and strategies for biological contaminations would be necessary and prioritized [25–28].

In this work, we demonstrated the development of an effective strategy for selectively eliminating biological contaminants and rescuing the cyanobacteria-based ethanol photosynthetic production. An ethanol-consuming contaminant infecting the outdoor cultivation system was identified and characterized. Based on physiological characterization of the ethanol-consuming contaminant, a pH-rising strategy was designed and adopted, which effectively inhibited growths of the contaminant and rescued ethanol production in the outdoor non-sterilized cultivation systems. Our work demonstrated the importance of effective contamination control strategies and techniques for large scale outdoor cultivations of the photosynthetic cell factories, and provided an inspiring paradigm for achieving targeted and effective solutions.

## Results

### Devastated photosynthetic ethanol production of an engineered cyanobacteria strain in non-sterilized outdoor cultivations

**Fig. 1:**
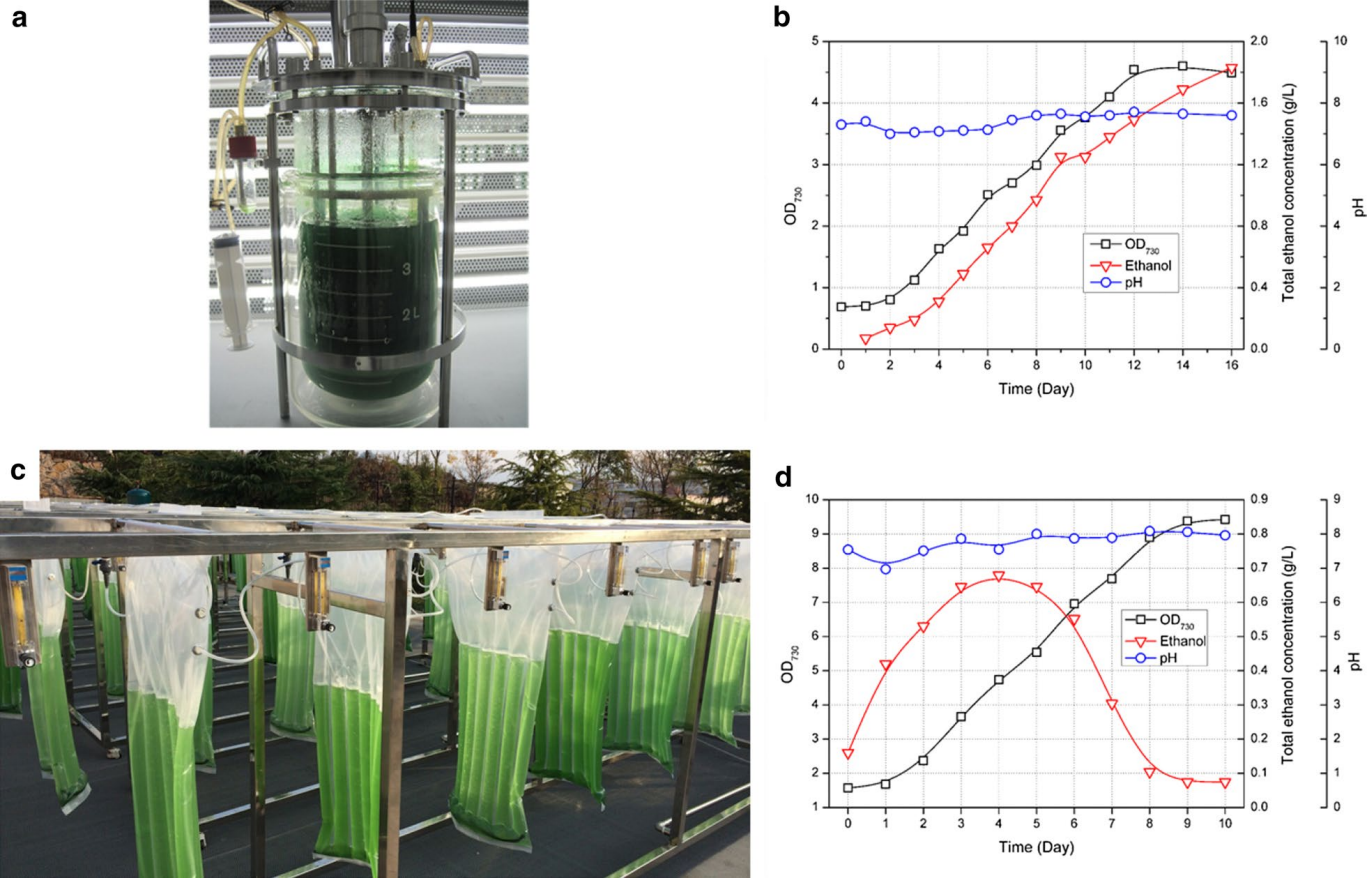
Ethanol photosynthetic production of an engineered cyanobacteria strain Syn-HZ24 in lab scale and in non-sterilized outdoor cultivations. **a** 3.5 L tank photobioreactor used for sterilized cultivation in lab. **b** Growth (*open square*), ethanol accumulation (*open triangle*) and pH (*open circle*) values of Syn-HZ24 sterilized cultivation in tank photobioreactor supplied with 5% CO_2_–air stream. **c** 6 L hanging membrane photobioreactors used for non-sterilized outdoor cultivations. **d** Growth (*open square*), ethanol accumulation (*open triangle*) and pH (*open circle*) values of the outdoor cultivations in hanging membrane photobioreactor supplied with 5% CO_2_–air stream

Previously, we have reported the development of an efficient ethanol-synthesizing cyanobacteria strain Syn-HZ24 (HZ24 hereafter for short) derived from *Synechocystis* sp. PCC6803. For achieving efficient ethanol photosynthetic production, *pdc* gene from *Zymomonas mobilis* (*Zmpdc*) encoding pyruvate decarboxylase and an endogenous gene *slr1192* encoding alcohol dehydrogenase II were overexpressed in *Synechocystis* sp. PCC6803, while the pathway for poly-β-hydroxybutyrate (PHB) synthesis was disrupted. Growths and ethanol synthesis capacities of HZ24 were evaluated in a lab-scale 5 L photobioreactor (with 3.5 L working volume, Fig. 1a), bubbled with 5% CO_2_–air stream under sterilized conditions. As shown in Fig. 1b, 1.8 g/L ethanol was obtained after 16 days cultivation with an OD_730_ of 4.5. Due to the influence of ethanol accumulation and increasing cell densities, the fastest growth was obtained during day 2 to day 6. During the ethanol synthesis process, pH values of the cultivation systems were maintained on the neutral or weak alkaline level, ranging from 7.0 to 8.0.

For scaling up the ethanol photosynthetic production process, we cultivated HZ24 in designated polyethylene membrane photobioreactors (MPBR) in outdoor environments (Fig. 1c). Working volume for each photobioreactor was 6 L, and 5% CO_2_–air stream was bubbled. Similar with the cultivation process in sterilized photobioreactors (Fig. 1b), pH values of the outdoor cultivation system were maintained around 8.0 during the whole process. However, ethanol accumulations in the outdoor cultivation systems were devastated. Ethanol concentration in the MPBR reached 0.67 g/L (0.6 g/L in the culture broth and 0.07 g/L in the recovery systems) after 4 days cultivation, and then sharply decreased to 0.07 g/L (0 g/L in the culture broth and 0.07 g/L in the recovery system) in the following 6 days, indicating the ethanol synthesis and accumulation were stopped since day 5 (Fig. 1d). Optical densities of the outdoor cultivations kept growing while ethanol accumulations were ceased and decreased, indicating that some biocontaminations might infect the cultivation system, inhibit the ethanol production or even consume the accumulated ethanol.

### Diagnosis of outdoor cultivation process and identification of the ethanol-consuming contaminant

Microscopic analysis revealed that the culture broths from non-sterilized cultivations were infected by bacilliform microorganisms. The more contaminants appeared in the broth, the more ethanol was consumed (Additional file 1: Figure S1), indicating a direct relationship between the biocontaminations and devastated ethanol production.

**Fig. 2:**
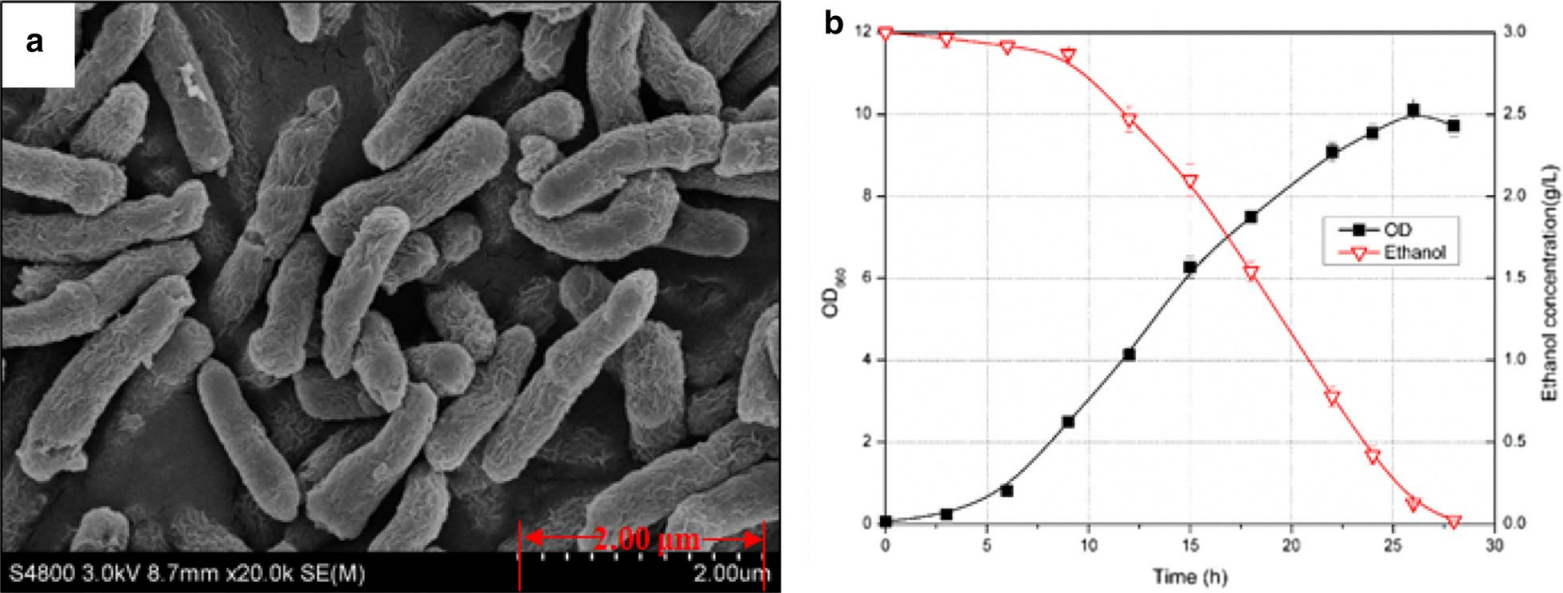
Characterization of *Pannonibacter phragmitetus* isolated from non-sterilized outdoor cultivations. **a** Scanning electron microphotograph of *Pannonibacter phragmitetus*. **b** Growth (*closed square*) and ethanol consumption analysis (*open triangle*) of *Pannonibacter phragmitetus* cells under rich medium. *Error bars* correspond to the standard deviation determined for three independent experiments

The bacilliform contaminant was isolated, purified, and identified. The 1388-bp-sized 16S rRNA sequence shows over 99% similarity with that of *Pannonibacter phragmitetus* [29]. Scanning electron microscopy analysis revealed that the contaminant was rod-shaped, with cell lengths of 1.5–2 μm and diameters of 0.2–0.3 μm (Fig. 2a).

To confirm the hypothesis that the decreased ethanol accumulations in the broths were caused by infection of the specific contaminant, we performed ethanol-consuming activities assays with the isolated *Pannonibacter phragmitetus* strain. As shown in Fig. 2b, the isolated and purified *Pannonibacter phragmitetus* strain could grow in LB medium and consume the supplemented ethanol. After 28 h of cultivation, 3 g/L ethanol was completely consumed, while OD_660_ of the broth reached up to 10, indicating that infection of the *Pannonibacter phragmitetus* strain in the outdoor cultivation systems should be responsible for the devastated ethanol production.

### High pH conditions inhibited growths and ethanol consumptions of the contaminant *Pannonibacter phragmitetus*

Photosynthetic-synthesized ethanol of the engineered cyanobacteria strain HZ24 was completely devastated by the infection of *Pannonibacter phragmitetus*; thus, development of a strict control strategy inhibiting growths and ethanol consumptions of the specific contaminant would be required for rescuing the ethanol production in non-sterilized outdoor cultivation systems. To explore selective strategies, we assayed the growths and ethanol-consuming capacities of the purified *Pannonibacter phragmitetus* under hypersaline and alkaline conditions.

**Fig. 3:**
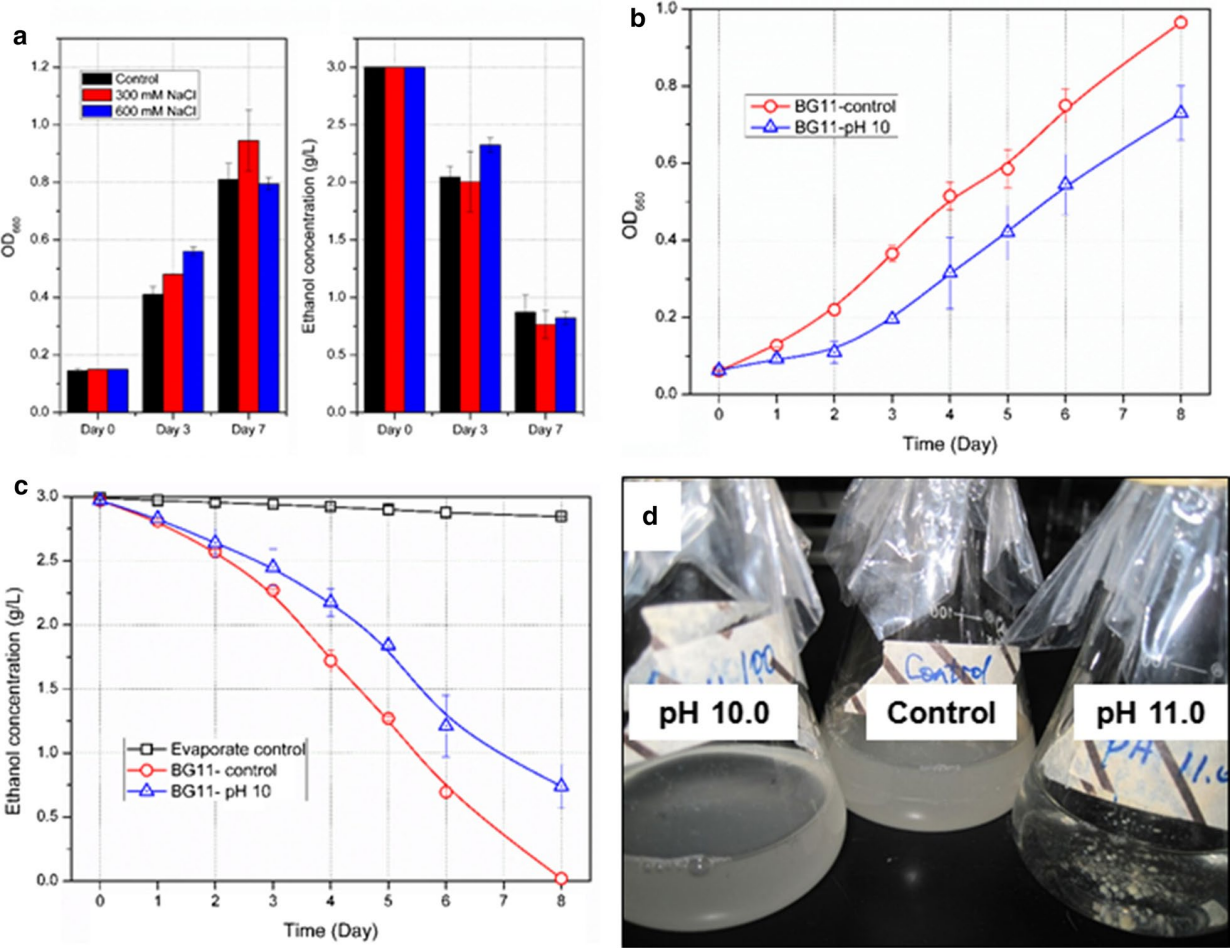
Effects of sodium chloride and high pH stress on growth and ethanol-consuming activities of *Pannonibacter phragmitetus*. **a** Growth (*left*) and ethanol consumption (*right*) of *Pannonibacter phragmitetus* in BG11 medium with 0 mM NaCl (shown in *black column*), 300 mM NaCl (shown in *red column*) and 600 mM NaCl (shown in *blue column*). **b** Growths of *Pannonibacter phragmitetus* in BG11 medium with original pH (*open circle*) and pH 10 (*open triangle*). **c** Ethanol consuming activities of *Pannonibacter phragmitetus* in BG11 medium with original pH (*open circle*) and pH 10 (*open triangle*), the natural evaporation (*open square*) was taken as a control.* Error bars* corresponded to the standard deviation determined from three independent experiments. **d** Growths of *Pannonibacter phragmitetus* cells cultivated in BG11 medium with original pH, pH 10 and pH 11.0 for 3 days

As shown in Fig. 3a, when NaCl concentrations in BG11 culture medium reached 600 mM, neither growths nor ethanol-consuming activities of *Pannonibacter phragmitetus* were inhibited, indicating hypersaline conditions might not be an acceptable strategy for controlling infection of *Pannonibacter phragmitetus*. In addition, *Pannonibacter phragmitetus* could grow in BG11 culture medium supplemented with ethanol independently with cyanobacteria indicated that ethanol could be absorbed and converted by this contaminant as sole carbon sources.

We further explored the inhibition effects of alkaline conditions on *Pannonibacter phragmitetus.* As shown in Fig. 3b and c, when pH value of the BG11 medium was elevated to 10.0, buffered by *N*-Cyclohexyl-3-aminopropanesulfonic acid, growths of *Pannonibacter phragmitetus* were significantly inhibited, and the ethanol consumption rates were decreased by 30%. When the pH value was further increased to 11.0, growths of *Pannonibacter phragmitetus* were completely ceased and the dead cells agglomerated at bottom of the flasks (Fig. 3d). *Pannonibacter phragmitetus* strains were first isolated from soda lake and identified as haloalkaliphilic bacterium [29]. While in recent years, groups of *Achromobacter* isolates with significant phenotypic divergences were also classified as *Pannonibacter phragmitetus* [30]. The newly isolated *Pannonibacter phragmitetus* strain in this work was enriched from neutral or weak alkaline cultivation environments with gradually increasing ethanol, and thus displayed quite different characteristics, showing alkali-sensitivity rather than alkali philicity. The ethanol-consuming contaminant *Pannonibacter phragmitetus* was sensitive to high pH conditions while the ethanol-synthesizing cyanobacteria strain was able to tolerate wide pH ranges, indicating that alkaline conditions might be an effective approach to rescue ethanol photosynthetic production for the outdoor cultivation systems.

### Infection of *Pannonibacter phragmitetus* in the ethanol photosynthetic production process could be inhibited by high pH conditions

Considering that the growths and ethanol consumptions of *Pannonibacter phragmitetus* could be inhibited by high pH conditions, we explored to control the infection of this contaminant in the process of ethanol photosynthetic production by a pH-rising strategy. In laboratory scale, we explored this strategy in 600 mL column photobioreactors and adopted a Bicarbonate-based Integrated Carbon Capture System (BICCS) to raise and maintain the pH values of the cultivation systems, as for which 180 mM NaHCO_3_ was supplied in the medium as main inorganic carbon sources while air steam would be bubbled for pH control and regulation. As a control without pH-rising system, 5% CO_2_ would be bubbled into the culture broths for providing carbon sources.

**Fig. 4:**
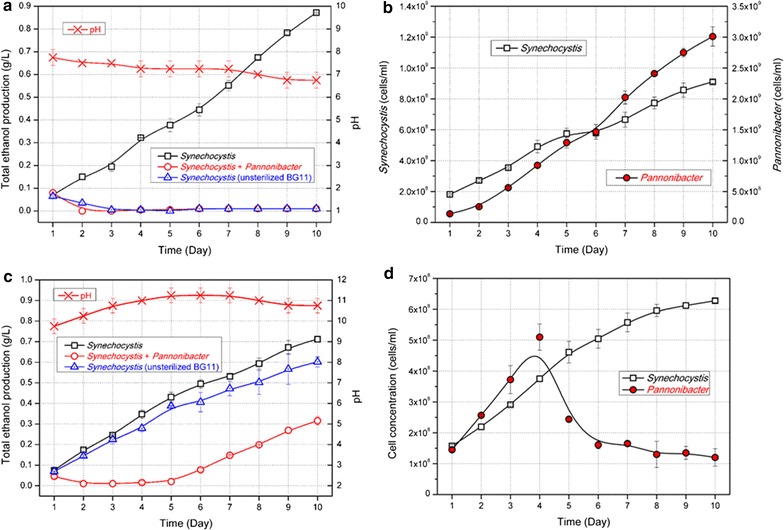
Evaluation of the contamination-controlling effects of BICCS based pH-rising strategy for ethanol photosynthetic production of Syn-HZ24. **a** Ethanol production of Syn-HZ24 cultivated in sterilized (*red open squares*), non-sterilized (*blue triangles*), and *Pannonibacter phragmitetus* inoculated BG11 culture medium bubbled with 5% CO_2_ as main carbon source. *Red crosses* denoted the pH values during the cultivation process. **b** CFU dynamics of *Synechocystis* (*open square*) and *Pannonibacter* (*closed circle*) cultivated in BG11 culture medium bubbled with 5% CO_2_–air steam without pH-rising strategy. **c** Ethanol production of Syn-HZ24 cultivated in sterilized (*red open squares*), non-sterilized (*blue triangles*), and *Pannonibacter phragmitetus* inoculated BG11 culture medium with BICCS-based pH-rising strategy.* Red crosses* denoted the pH values during the cultivation processes. pH values were allowed to increase to 11 physiologically and then constantly maintained at 11 with 180 mM NaHCO_3_ and air stream. **d** CFU dynamics of *Synechocystis* (*open square*) and *Pannonibacter* (*closed circle*) cultivated in BG11 culture medium with the BICCS-based pH-rising strategy. pH values of the cultivation process were maintained in the range from 10 to 11

For mimics of the infected cultivations, purified *Pannonibacter phragmitetus* cells would be artificially inoculated in sterilized BG11 culture medium. As shown in Fig. 4a, when no pH-rising strategy was adopted, about 0.9 g/L ethanol was obtained in the cultivation systems with sterilized BG11 culture medium, while the inoculation of *Pannonibacter phragmitetus* cells completely devastated the ethanol production, similar with the cultivations with non-sterilized culture medium, meaning that infection of the isolated *Pannonibacter phragmitetus* strain could be the main reason responsible for devastation of photosynthetic ethanol production in this case. During the 10-day cultivation, pH values of the cultivations were maintained in the range from 7.0 to 8.0, under which concentrations of *Pannonibacter phragmitetus* cells artificially inoculated into the systems increased from 1.4–1.5 × 10^8^ to 2.8–3.2 × 10^9^ cells/mL, while cell concentrations of HZ24 were increased from 1.6–1.8 × 10^8^ to 9 × 10^8^ cells/mL, much slower than that of *Pannonibacter phragmitetus* (Fig. 4b).

In comparisons, when the pH-rising strategy was adopted and pH values of the culture broth were maintained ranging from 10.0 to 11.0, ethanol productions in non-sterilized cultivations and in *Pannonibacter phragmitetus* inoculated cultivations were both recovered (Fig. 4c). After 10-day cultivation, ethanol concentration in the sterilized systems reached 0.7 g/L, while that in non-sterilized systems was 0.6 g/L, indicating that the infection of *Pannonibacter phragmitetus* in non-sterilized cultivation systems was significantly inhibited by the high pH conditions. As for cultivations with artificially inoculated *Pannonibacter phragmitetus*, ethanol synthesis was retarded in the first 5 days, while in the following 5 days comparable ethanol productivity were recovered, achieving a final ethanol concentration of 0.3 g/L. The dynamics of ethanol production was in according with the cell concentrations fluctuations (Fig. 4d), as for which cells of *Pannonibacter phragmitetus* kept growing, and reached a peak (from about 1.4 × 10^8^ to 5 × 10^8^ cells/mL) at day 4, and then sharply decreased in the following 6 days (to 1.1 × 10^8^ cells/mL), while concentrations of HZ24 cells kept growing in the 10-day cultivation processes from 1.5 × 10^8^ to 6.1 × 10^8^ cells/mL. The retarded ethanol production in artificially inoculated cultivations might be a result of the higher cell concentrations of *Pannonibacter phragmitetus*, which required a longer time to be completely inhibited. In addition, carbon partitioning ratios calculations revealed that cultivations taking NaHCO_3_ as carbon sources did not influence ethanol-synthesizing capacities of the HZ24 strain. About 39.6% of the fixed carbon was directed into ethanol synthesis in the pH-rising system, while that in 5% CO_2_ pumped system was 38.7%, meaning that the intracellular ethanol-synthesizing capacities on global cellular levels were not inhibited by high pH conditions.

### Rescuing ethanol photosynthetic production in non-sterilized outdoor cultivations with the BICCS-derived pH-rising strategy

**Fig. 5:**
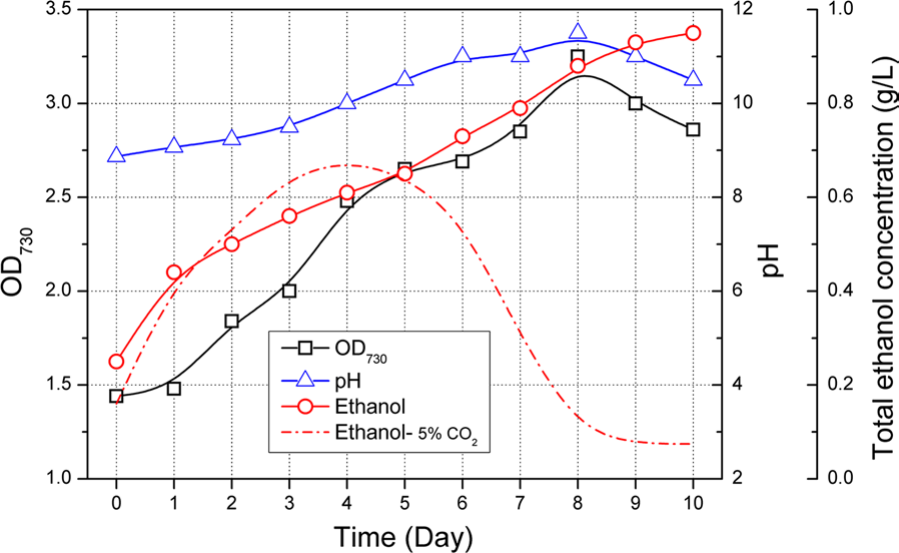
Growth and ethanol production of Syn-HZ24 in non-sterilized outdoor cultivation systems with BICCS-based pH-rising strategy. *Open square* denoted cell growth; *open triangle* denoted pH values; *open circle* denoted ethanol accumulations*; red dotted line* denoted the ethanol production of Syn-HZ24 non-sterilized outdoor cultivation without pH-rising strategy

The BICCS-derived pH-rising strategy proved to be effective for controlling infections of the ethanol-consuming contaminant *Pannonibacter phragmitetus* in laboratory scale non-sterilized cultivations or artificially contaminant-inoculated cultivations, and we further explored to rescue ethanol photosynthetic production in outdoor non-sterilized environments. The BICCS was also adopted to maintain an alkaline condition (pH values around 11.0) in the culture broths of designated polyethylene membrane photobioreactors (MPBR). As shown in Fig. 5, in the first 6-day cultivations, pH values of the broth were slightly increased from 9.0 to 11.0, and maintained at this level for the following 4 days. During this process, OD_730_ grew to a peak value of 3.2 in day 8. Ethanol production continued and accumulated to a final titer of 0.95 g/L. In comparisons, ethanol concentration in cultivation systems taking 5% CO_2_ as sole carbon source (without pH-rising) reached a peak value of 0.6 g/L in day 4, and then be consumed completely in the following 6 days. Comparing with ethanol photosynthetic production of HZ24 in column photobioreactors under stable lab conditions (with continuous light intensities and constant temperatures), the carbon partitioning ratio in outdoor MPBR was significantly decreased (both the productivities and carbon partitioning ratios), which might resulted from the fluctuating environment conditions (sun light intensities, day-night cycles, and changing temperatures). In summary, our results confirmed that the pH-rising strategy successfully inhibited the infection of ethanol-consuming biocontaminant, and rescued the ethanol photosynthetic production in outdoor non-sterilized environments.

## Discussion

As for large-scale cultivation of microalgae and cyanobacteria, biocontaminations infection would overcome or kill the photosynthetic microorganisms, and inhibit or devastate the accumulations of cellular biomass or target bioproducts [31–33]. Thus, development of effective and selective contamination-control strategies is of significant importance for the scaling up processes. However, photosynthetic cell factories were usually derived from diverse cyanobacteria or microalgae species with diversified physiological or metabolism characteristics, and the products production processes would influence and interact with the cultivation environments, thus leading to characteristic contamination patterns for specific scaling up processes. In this work, we demonstrated an effective paradigm for “diagnosing and healing” the ethanol photosynthetic production with an engineered cyanobacteria strain under non-sterilized outdoor cultivations. Comparing with previously reported cases and patterns [21, 28], the main biocontaminations infecting our cultivation systems did not influence the growths or photosynthetic production of the engineered cyanobacteria strain but directly consume ethanol, the photosynthetic products. Physiological and growths analysis revealed that the ethanol-consuming contaminant, belonging to *Pannonibacter phragmitetus*, was sensitive to alkaline stress at pH 11, while cyanobacteria strain applied for ethanol synthesis was alkaline tolerant, and thus a pH-rising strategy was designed and adopted to selectively inhibit or even kill the contaminants. Usually the outdoor cultivation systems under non-sterilized conditions would be infected and influenced by more than one contaminant [24]. As an outdoor large scale cultivation system under non-sterilized conditions, infections of bio-contaminants could be picked up through multi ways, including bubbled air, the water used for culture medium preparations, and the photobioreactors. In fact, we have also ever discovered infection of other contamination microorganisms rather than *Pannonibacter phragmitetus*, while such infections were neither frequent nor stable. When the cultivation systems were not infected by *Pannonibacter phragmitetus* but other contaminations, ethanol production would not be significantly influenced. While when *Pannonibacter phragmitetus* inflected the cultivation system, other microorganism could hardly be identified by microscopic observation or agar plates cultivations. At least in this study a specific contaminant *Pannonibacter phragmitetus* was the main threat for successful ethanol production, and influences of the other potential contaminants were limited. The influence of pH-rising strategy on other contaminants was currently unknown, while it indeed solved the essential problem restricting ethanol photosynthetic production in the current system shown here.

Lots of cyanobacteria strains have the capacities of growing at harsh alkaline environments [34, 35], and it has also been reported that photosynthesis activities of *Synechocystis* sp. PCC6803 would not be influenced by high pH values of the medium [21, 36], indicating that the pH-rising strategy might be able to be adopted as a common approach for contamination control in scaling up processes of cyanobacteria. Thus, it is of great importance to develop an effective and convenient pH-rising strategy with reduced inhibition on cyanobacteria strains. Previously, it has been reported that for minimizing the stress to cyanobacteria cells, pH values of the cyanobacteria culture broths would physiologically rise to 11.0, while after that NaOH and CO_2_ was separately and stepwise supplemented in dark conditions and in light conditions, respectively, to maintain the alkaline conditions [21]. Taking the requirements and restrictions in large-scale cultivations into consideration, we adopted a Bicarbonate-based Integrated Carbon Capture System [37–39] as the pH-rising strategy, as for which bicarbonate would be taken as the sole carbon source, leading to a high alkalinity of the culture medium. As for the BICCS, CO_2_ would be captured as bicarbonate and absorbed by cyanobacteria cells during the pH controlling process, while the carbonate regenerated by the culture process would be used as an absorbent to capture more CO_2_ [39]. Comparing with the previously adopted strategy using NaOH and CO_2_, this pH-rising strategy could not only minimize the stress of the cyanobacteria cells but also reduce the carbon capture costs, making it feasible and attractive for industrial applications.

Comparing with the contaminant *Pannonibacter phragmitetus*, cyanobacteria strain used in this work show better adaptability to alkaline conditions, and that was the basis for designing and adopting the pH-rising strategy. However, it was also noteworthy that growths and ethanol synthesis of the engineered cyanobacteria strain was also inhibited under the extreme alkaline conditions, ethanol production decreased by about 22% from 0.9 to 0.7 g/L, while carbon partitioning ratios were maintained (Fig. 4). The retarded growths and production performances might be caused by the increased demands for energy and materials to support HCO_3_^−^ absorption and energy maintaining [33, 40]. For further optimizing the ethanol photosynthetic production in outdoor non-sterilized conditions, robustness of the engineered cyanobacteria strains facing alkaline stresses should be enhanced, which could be achieved by evolutionary approaches [21, 41, 42] or introduction of stress-tolerant devices [43–45].

## Conclusions

In this work, we diagnosed and rescued the ethanol photosynthetic production with an engineered *Synechocystis* strains under non-sterilized outdoor cultivation processes. In the scaling up process of ethanol photosynthetic production with engineered *Synechocystis* sp. PCC 6803, infection of an ethanol-consuming contaminant *Pannonibacter phragmitetus* completely ceased and devastated ethanol accumulations. Based on growth and physiology analysis of the contaminant, we adopted a Bicarbonate-based Integrated Carbon Capture System to maintain an alkaline conditions (pH 11.0) in the cultivation systems, which successfully inhibited the infection and recovered the photosynthetic ethanol production. In summary, we demonstrated a paradigm for developing an effective strategy for selectively eliminating biological contaminants and rescuing the cyanobacteria-based photosynthetic production of biofuels or biochemicals.

## Methods

### Cultivations of *Synechocystis* strain HZ24

Ethanol-synthesizing strain HZ24 derived from S*ynechocystis* sp. PCC 6803 [20] was pre-cultivated in 500 mL Erlenmeyer flasks containing 300 ml BG11 medium [46] with constant 50 μE/m^2^/s^1^ white light, continuously sparged with ambient air at 30 °C. Antibiotics would be supplemented when required.

Sharp-bottom column photobioreactors (total length 580 mm, diameter 50 mm, working volume 600 mL) were used for HZ24 cultivation with sterilized, non-sterilized, and *Pannonibacter phragmitetus* inoculated BG11 medium. Cool white lamps with constant fixed photon flux density of 100 μE/m^2^/s^1^ were used for providing lights, while the temperature was maintained at 30 °C. *Synechocystis* cells were grown to the exponential phase and harvested by centrifugation, resuspended in fresh BG11 medium and transferred to column photobioreactors bubbled with 5% CO_2_–air (for cultivation without pH-rising strategy) or resuspended in fresh BG11 medium added with 180 mM NaHCO_3_ and transferred to column photobioreactors bubbled with air (for cultivation under high pH values). Flow rates of air or CO_2_–air mixture were maintained at 0.1 vvm.

5 L photobioreactor (Biostat Bplus, Sartorius) with working volume of 3.5 L was used for cultivation of HZ24 under sterilized condition (Fig. 1a). CO_2_–air mixture gas (5% CO_2_) with flow rates of 0.1 vvm was bubbled into the culture medium to provide carbon sources. Cultivations were performed at 30 °C, with agitation speeds of 100 rpm and constant fixed photon flux density of 100 μE/m^2^/s^1^ light.

Hanging polyethylene membrane photobioreactors (MPBR) with working volume of 6 L were used for outdoor cultivation (Fig. 1c). The cultivation compartment of MPBR consisted of 7 parallel tubes (length 640 mm, diameter 40 mm) connected by a horizontal tube (length 340 mm, diameter 40 mm). The culture broths were agitated by continuous air flow with a rate of 0.1 vvm through bubble diffusers placed at bottoms of MPBR. The environmental temperature ranged from 28 to 40 °C during the day time, and from 18 to 25 °C in night. No artificial light was supplied during the whole outdoor cultivation processes.

Ethanol carried by the air steam would be recovered as previously introduced (Additional file 1: Figure S2) [20].

### Cultivation of *Pannonibacter phragmitetus*

*Pannonibacter phragmitetus* cells were pre-cultivated in 100 mL flasks containing 30 ml Luria–Bertani (LB) medium [47] or BG11 medium, incubated on a rotary shaker at 30 °C and 200 rpm.

Growth and ethanol-consuming assays under normal or stressful conditions (hypersaline or alkaline) were performed in 250 mL shake flasks containing 100 mL LB or BG11 culture medium, at 30 °C and 200 rpm. For calculating ethanol-consuming capacities, 3 g/L would be supplemented in the culture medium. For hypersaline stressful conditions, NaCl would be added to a final concentration of 600 mM. For alkaline stressful conditions, *N*-Cyclohexyl-3-aminopropanesulfonic acid was used as high pH buffering agent when pH values of the culture medium were adjusted to 10.0 or 11.0.

### Growth and ethanol synthesis calculations

Samples from flasks or bioreactors were collected at 1-day intervals for OD_730_ and ethanol concentration determinations. OD730 was used to calculate biomass accumulation.

Ethanol concentration in bioreactor and recovery bottles were determined separately and added to calculate the total ethanol titer. As for samples from bioreactors, culture broth was centrifuged at 10,000*g* for 2 min, and the supernatant would be used for assay. Ethanol concentration was determined with a SBA-40C biosensor analyzer (Shandong Academy of Sciences, China) equipped with the ethanol oxidase immobilized membrane [20].

### CFU analysis for *Pannonibacter phragmitetus* and HZ24

Cell numbers of *Pannonibacter phragmitetus* and HZ24 cultured in the artificial pathogen-inoculated cultivation system were determined by monitoring CFU on the plate count agar plates by standard procedures. Briefly, culture broth samples were collected daily collected from the pathogen-inoculated cultivation system, stepwise diluted by 10^5^–10^8^ folds, and then spread on LB agar plates and BG11 agar plates. LB plates would be incubated on at 30 °C for 48 h, while BG11 plates would be incubated at 30 °C with constant 50 μE/m^2^/s^1^ white light for 4–7 days. After incubation, colonies of *Pannonibacter phragmitetus* and HZ24 would be distinguished and counted for cell number calculations. Antibiotics would be added into the agar plates as required.

## Additional file

**Additional file 1: Figure S1.** Microscopic analysis of the outdoor non-sterilized cultivation system for photosynthetic production of ethanol. **Figure S2.** Structure schematic of the ethanol recovery system for the MPBR system. **Figure S3.** Total ethanol production and distribution of Syn-HZ24 cultivated in MPBR under non-sterilized outdoor conditions.

## Abbreviations

KmR: kanamycin resistance gene
NADPH: reduced nicotinamide adenine dinucleotide phosphate
OD: optical density
PBR: photobioreactor
MPBR: membrane photobioreactors
PDC: pyruvate decarboxylase
SpR: spectinomycin resistance gene
BICCS: Bicarbonate-based Integrated Carbon Capture System

## References

[1] Kennes D, Abubackar HN, Diaz M, Veiga MC, Kennes C (2016). Bioethanol production from biomass: carbohydrate vs syngas fermentation. J Chem Technol Biot..

[2] Keasling JD, Chou H (2008). Metabolic engineering delivers next-generation biofuels. Nat Biotechnol.

[3] Thangavelu SK, Ahmed A, Ani FN (2016). Review on bioethanol as alternative fuel for spark ignition engines. Renew Sustain Energy Rev.

[4] Tabah B, Pulidindi IN, Chitturi VR, Arava LMR, Gedanken A (2016). Utilization of solar energy for continuous bioethanol production for energy applications. RSC Adv..

[5] Agarwal AK (2007). Biofuels (alcohols and biodiesel) applications as fuels for internal combustion engines. Prog Energ Combust..

[6] Weber C, Farwick A, Benisch F, Brat D, Dietz H, Subtil T (2010). Trends and challenges in the microbial production of lignocellulosic bioalcohol fuels. Appl Microbiol Biotechnol.

[7] Rude MA, Schirmer A (2009). New microbial fuels: a biotech perspective. Curr Opin Microbiol.

[8] Himmel ME, Ding SY, Johnson DK, Adney WS, Nimlos MR, Brady JW (2007). Biomass recalcitrance: engineering plants and enzymes for biofuels production. Science.

[9] Lu X (2010). A perspective: photosynthetic production of fatty acid-based biofuels in genetically engineered cyanobacteria. Biotechnol Adv.

[10] Angermayr SA, Hellingwerf KJ, Lindblad P, de Mattos MJT (2009). Energy biotechnology with cyanobacteria. Curr Opin Biotechnol.

[11] Hellingwerf KJ, de Mattos MJT (2009). Alternative routes to biofuels: light-driven biofuel formation from CO_2_ and water based on the ‘photanol’ approach. J Biotechnol.

[12] Gudmundsson S, Nogales J (2015). Cyanobacteria as photosynthetic biocatalysts: a systems biology perspective. Mol BioSyst.

[13] Waterbury JB, Watson SW, Guillard RRL, Brand LE (1979). Widespread occurrence of a unicellular, marine, planktonic, cyanobacterium. Nature.

[14] Vermaas W (1996). Molecular genetics of the cyanobacterium *Synechocystis* sp. PCC 6803: principles and possible biotechnology applications. J Appl Phycol.

[15] Oliver JW, Atsumi S (2014). Metabolic design for cyanobacterial chemical synthesis. Photosynth Res.

[16] Deng MD, Coleman JR (1999). Ethanol synthesis by genetic engineering in cyanobacteria. Appl Environ Microbiol.

[17] Dexter J, Armshaw P, Sheahan C, Pembroke JT (2015). The state of autotrophic ethanol production in Cyanobacteria. J Appl Microbiol.

[18] Dexter J, Fu PC (2009). Metabolic engineering of cyanobacteria for ethanol production. Energy Environ Sci.

[19] Luan G, Qi Y, Wang M, Li Z, Duan Y, Tan X (2015). Combinatory strategy for characterizing and understanding the ethanol synthesis pathway in cyanobacteria cell factories. Biotechnol Biofuels.

[20] Gao ZX, Zhao H, Li ZM, Tan XM, Lu XF (2012). Photosynthetic production of ethanol from carbon dioxide in genetically engineered cyanobacteria. Energy Environ Sci.

[21] Touloupakis E, Cicchi B, Benavides AMS, Torzillo G (2016). Effect of high pH on growth of *Synechocystis* sp. PCC 6803 cultures and their contamination by golden algae (*Poterioochromonas* sp.). Appl Microbiol Biot..

[22] Zemke PE, Sommerfeld MR, Hu Q (2013). Assessment of key biological and engineering design parameters for production of Chlorella zofingiensis (Chlorophyceae) in outdoor photobioreactors. Appl Microbiol Biotechnol.

[23] Carney LT, Lane TW (2014). Parasites in algae mass culture. Front Microbiol..

[24] Elena Kazamia SA-C, Chris Abell, Alison G. Smith. Designing consortia to increase productivity of ethanol-producing cyanobacteria. http://www.dema-etoh.eu/admin/common/files/1417091681_ekazamia-poster-bielefeld2014-v3.pdf.

[25] Rego D, Redondo LM, Geraldes V, Costa L, Navalho J, Pereira MT (2015). Control of predators in industrial scale microalgae cultures with pulsed electric fields. Bioelectrochemistry.

[26] Day JG, Slocombe SP, Stanley MS (2012). Overcoming biological constraints to enable the exploitation of microalgae for biofuels. Bioresour Technol.

[27] Simkovsky R, Effner EE, Iglesias-Sanchez MJ, Golden SS (2016). Mutations in novel lipopolysaccharide biogenesis genes confer resistance to amoebal grazing in *Synechococcus elongatus*. Appl Environ Microbiol.

[28] Simkovsky R, Daniels EF, Tang K, Huynh SC, Golden SS, Brahamsha B (2012). Impairment of O-antigen production confers resistance to grazing in a model amoeba-cyanobacterium predator-prey system. Proc Natl Acad Sci USA.

[29] Borsodi AK, Micsinai A, Kovacs G, Toth E, Schumann P, Kovacs AL (2003). *Pannonibacter phragmitetus* gen. nov., sp. nov., a novel alkalitolerant bacterium isolated from decomposing reed rhizomes in a Hungarian soda lake. Int J Syst Evol Microbiol.

[30] Holmes B, Segers P, Coenye T, Vancanneyt M, Vandamme P (2006). *Pannonibacter phragmitetus*, described from a Hungarian soda lake in 2003, had been recognized several decades earlier from human blood cultures as Achromobacter groups B and E. Int J Syst Evol Micr..

[31] Wang H, Zhang W, Chen L, Wang J, Liu T (2013). The contamination and control of biological pollutants in mass cultivation of microalgae. Bioresour Technol.

[32] Richmond A (1992). Open systems for the mass-production of Photoautotrophic microalgae outdoors—physiological principles. J Appl Phycol.

[33] Touloupakis E, Cicchi B, Torzillo G (2015). A bioenergetic assessment of photosynthetic growth of *Synechocystis* sp. PCC 6803 in continuous cultures. Biotechnol Biofuels.

[34] McGinn PJ, Dickinson KE, Bhatti S, Frigon JC, Guiot SR, O’Leary SJ (2011). Integration of microalgae cultivation with industrial waste remediation for biofuel and bioenergy production: opportunities and limitations. Photosyn Res..

[35] Pikuta EV, Hoover RB, Tang J (2007). Microbial extremophiles at the limits of life. Crit Rev Microbiol.

[36] Summerfield TC, Sherman LA (2008). Global transcriptional response of the alkali-tolerant cyanobacterium *Synechocystis* sp. strain PCC 6803 to a pH 10 environment. Appl Environ Microbiol.

[37] Chi Z, Elloy F, Xie Y, Hu Y, Chen S (2014). Selection of microalgae and cyanobacteria strains for bicarbonate-based integrated carbon capture and algae production system. Appl Biochem Biotechnol.

[38] Chi Z, Xie Y, Elloy F, Zheng Y, Hu Y, Chen S (2013). Bicarbonate-based integrated carbon capture and algae production system with alkalihalophilic cyanobacterium. Bioresour Technol.

[39] Chi Z, O’Fallon JV, Chen S (2011). Bicarbonate produced from carbon capture for algae culture. Trends Biotechnol.

[40] Giordano M, Beardall J, Raven JA (2005). CO2 concentrating mechanisms in algae: mechanisms, environmental modulation, and evolution. Annu Rev Plant Biol.

[41] Luan G, Cai Z, Li Y, Ma Y (2013). Genome replication engineering assisted continuous evolution (GREACE) to improve microbial tolerance for biofuels production. Biotechnol Biofuels.

[42] Sauer U (2001). Evolutionary engineering of industrially important microbial phenotypes. Adv Biochem Eng Biotechnol.

[43] Luan G, Dong H, Zhang T, Lin Z, Zhang Y, Li Y (2014). Engineering cellular robustness of microbes by introducing the GroESL chaperonins from extremophilic bacteria. J Biotechnol.

[44] Lin Z, Zhang Y, Wang J (2013). Engineering of transcriptional regulators enhances microbial stress tolerance. Biotechnol Adv.

[45] Pan J, Wang J, Zhou ZF, Yan YL, Zhang W, Lu W (2009). IrrE, a global regulator of extreme radiation resistance in *Deinococcus radiodurans*, enhances salt tolerance in *Escherichia coli* and *Brassica napus*. PLoS ONE.

[46] Rippka R, Deruelles J, Waterbury JB, Herdman M, Stanier RY (1979). Generic assignments, strain histories and properties of pure cultures of Cyanobacteria. J Gen Microbiol.

[47] Wang YY, Peng B, Yang ZH, Tang CJ, Chen YH, Liao Q (2014). Treatment of Cr(VI) contaminated water with *Pannonibacter phragmitetus* BB. Environ Earth Sci..

