# Supplementary material for: Rescuing ethanol photosynthetic production of cyanobacteria in non-sterilized outdoor cultivations with a bicarbonate-based pH-rising strategy
Source: Biotechnol Biofuels. 2017 Apr 14;10:93. doi: 10.1186/s13068-017-0765-5 (PMC5391583; doi:10.1186/s13068-017-0765-5)
Supplement: Supplementary file 1 — Additional file 1: Figure S1. Microscopic analysis of the outdoor non-sterilized cultivation system for photosynthetic production of ethanol. Figure S2. Structure schematic of the ethanol recovery system for the MPBR system. Figure S3. Total ethanol production and distribution of Syn-HZ24 cultivated in MPBR under non-sterilized outdoor conditions. [file 13068_2017_765_MOESM1_ESM.docx]

**
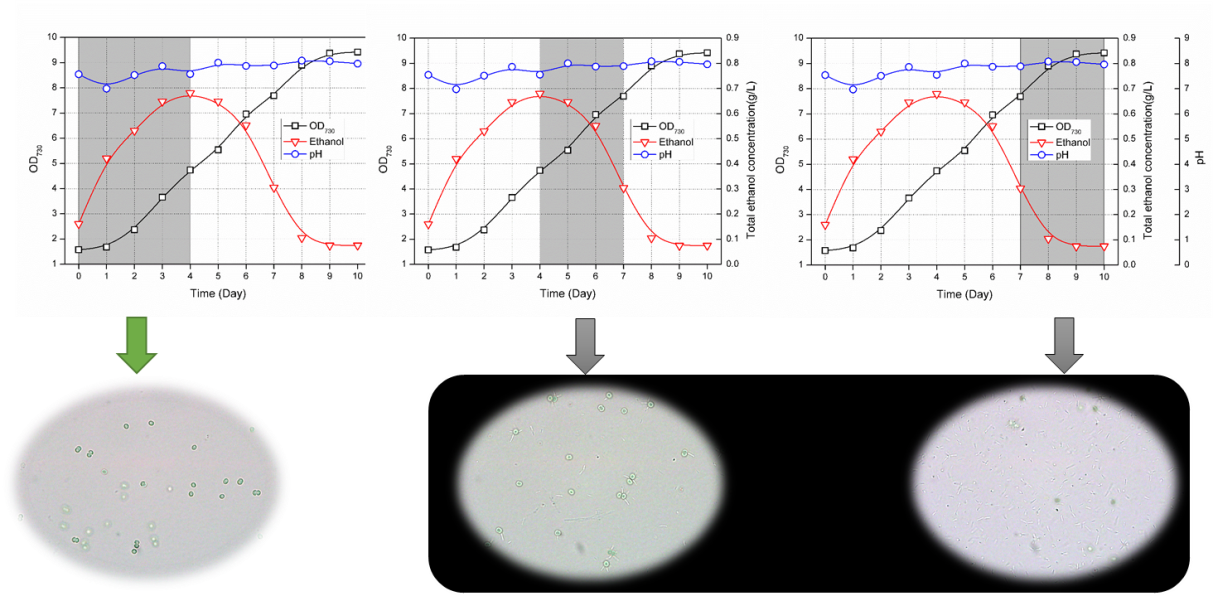
**

Figure S1. Microscopic analysis of the outdoor non-sterilized cultivation system for photosynthetic production of ethanol. In the initial 4 days of cyanobacteria cultivation when ethanol synthesis and accumulation was normal, no bio-contaminants were observed through microscope. While in the following 6 days during the process that ethanol was rapidly consumed, the rod-shaped contaminants appeared and occupied the cultivation.

**
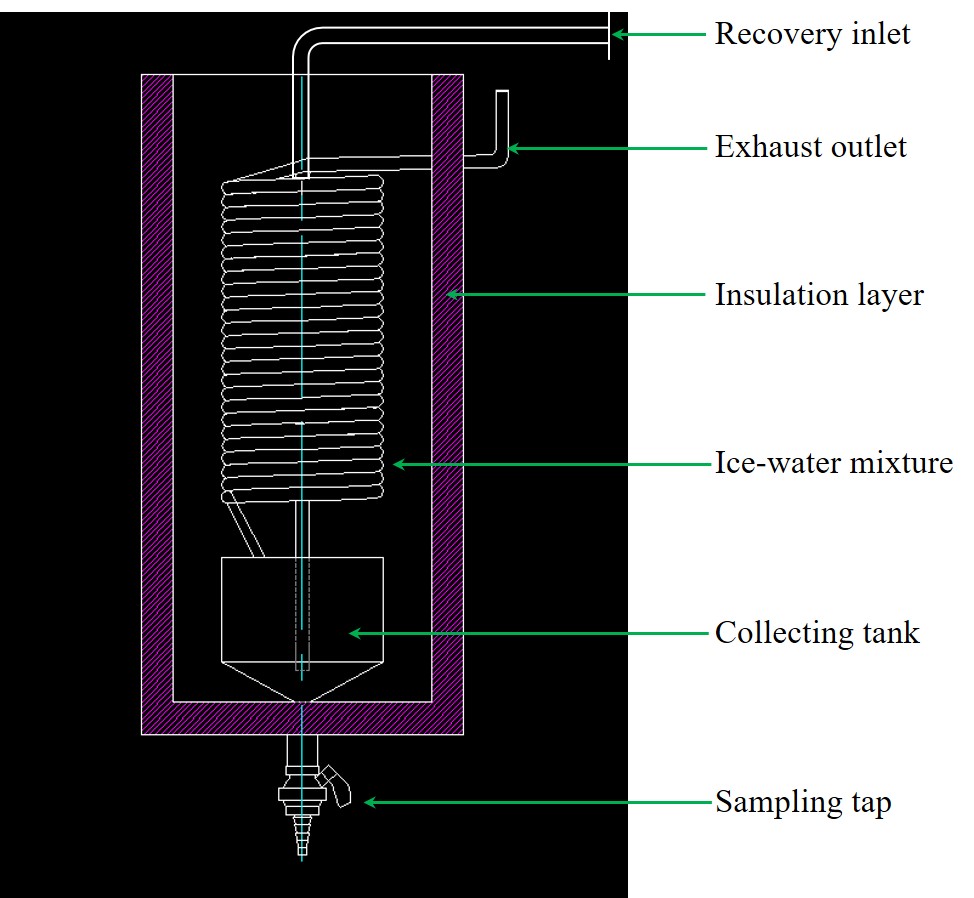
**

Figure S2. Structure schematic of the ethanol recovery system for the MPBR system. Ethanol recovery system was connected with the photobioreactors due to the volatility of ethanol. The recovery inlet was connected with the gas outlet of the photobioreactor. The ethanol-containing gas was led into the collecting tank directly which was filled with 300-500 ml water. And the exhaust gas was emitted through a serpentine outlet. The recovery inlet, collecting tank and the serpentine exhaust outlet were submerged in the ice-water mixture with a jacket structure (insulation layer) for insulation. And the sampling tap was used for collection of the ethanol recovered solution.


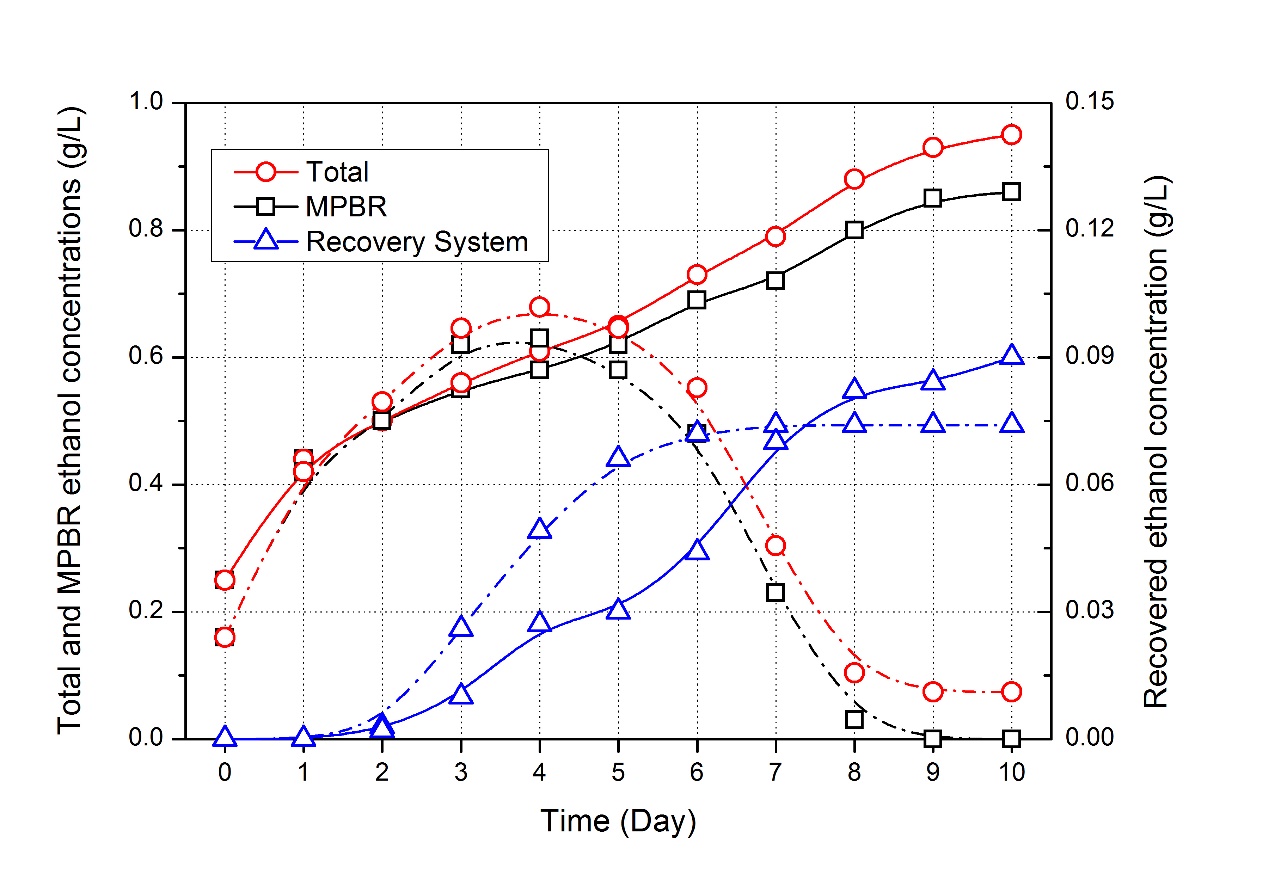


Figure S3. Total ethanol production and distribution of Syn-HZ24 cultivated in MPBR under non-sterilized outdoor conditions with (solid lines) or without (dotted lines) the BICCS based pH-rising strategy. Open circles denoted total ethanol concentrations; open squares denoted ethanol concentrations produced in MPBR; open triangles denoted the recovered ethanol concentrations.
